# Twenty-four-hour ambulatory, but not clinic blood pressure associates with leptin in young adults with overweight or obesity: The African-PREDICT study

**DOI:** 10.1038/s41440-023-01477-7

**Published:** 2023-10-23

**Authors:** Elandi van Niekerk, Shani Botha-Le Roux, Catharina M. C. Mels, Mariette Swanepoel, Christian Delles, Paul Welsh, Ruan Kruger

**Affiliations:** 1 Hypertension in Africa Research Team (HART), North-West University, Potchefstroom, South Africa; 2 Medical Research Council: Research Unit for Hypertension and Cardiovascular Disease, North-West University, Potchefstroom, South Africa; 3 Physical activity, Sport and Recreation (PhASRec), North-West University, Potchefstroom, South Africa; 4 School of Cardiovascular and Metabolic Health, University of Glasgow, Glasgow, UK

**Keywords:** Blood pressure, Inflammation, Leptin, Obesity, Young adults

## Abstract

Hypertension and obesity are known pro-inflammatory conditions, and limited studies explored various blood pressure modalities and inflammatory markers in young adults with overweight or obesity (OW/OB). We assessed the relationship of clinic and 24 h ambulatory blood pressure with an array of inflammatory markers in young adults with OW/OB. This cross-sectional study included women and men of Black and White ethnicity (*n* = 1194) with a median age of 24.5 ± 3.12 years. Participants were divided into normal weight and OW/OB groups according to body mass index. Clinic and 24 h ambulatory systolic and diastolic blood pressure were measured. Inflammatory markers included leptin, interleukin-6, interleukin-8, tumour necrosis factor-α, adiponectin, interleukin-10, and C-reactive protein. After adjustments for age, sex, and ethnicity, the OW/OB group had higher blood pressure and an overall worse inflammatory profile compared to the normal weight group (all *p* ≤ 0.024). In the OW/OB group, 24 h systolic (*r* = 0.22; *p* < 0.001) and diastolic blood pressure (*r* = 0.28; *p* < 0.001) correlated with leptin, independent of age, sex, and ethnicity. In fully adjusted regression models, 24 h systolic blood pressure (adj.R^2^ = 0.25; *β* = 0.28; *p* = 0.035) and diastolic blood pressure (adj.R^2^ = 0.10; *β* = 0.32; *p* = 0.034), associated with leptin in the OW/OB group and significance remained with additional adjustments for visceral adiposity index. Twenty-four-hour ambulatory, but not clinic blood pressure, is related to leptin in young adults with OW/OB. Leptin shows a stronger relationship with adiposity when compared to other inflammatory markers and may play a role in subcutaneous adiposity-related increased blood pressure.

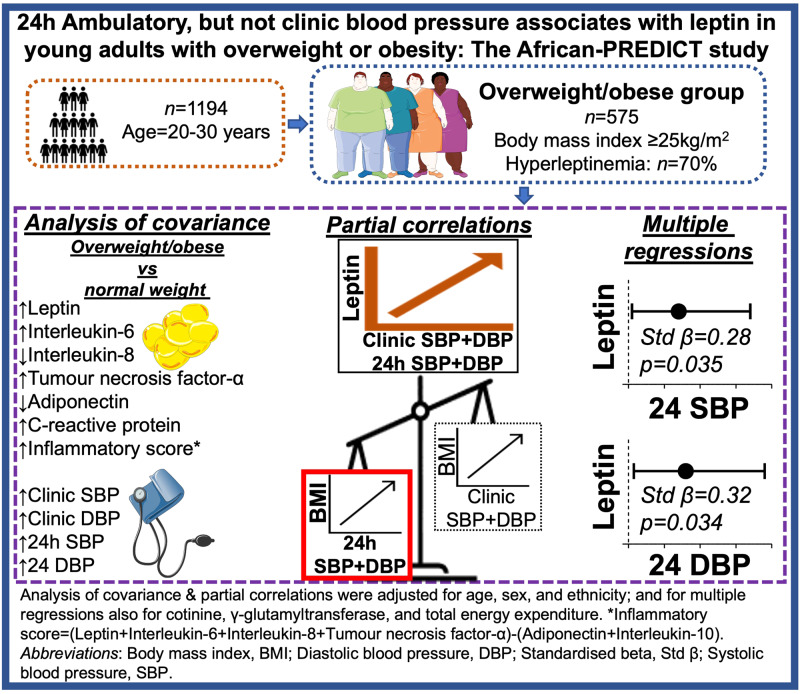

## Introduction

Hypertension is the leading global risk factor of attributable deaths [1], with obesity being a major risk factor for hypertension [2]. Hypertension and obesity are both pro-inflammatory conditions [3], and higher levels of inflammation show a causal link with increased blood pressure (BP) [4]. Adiposity-related pro-inflammatory markers such as leptin, interleukin-6 (IL-6), and tumour necrosis factor-α (TNF-α), and lower levels of anti-inflammatory markers, such as adiponectin play a role in the development of hypertension [5]. A study in hypertensive (*n* = 112; 66 ± 10 years old) and resistant hypertensive adults (*n* = 112; 58 ± 10 years old) indicated that an integrated inflammatory score (consisting of leptin, IL-6, interleukin-8 (IL-8), TNF-α, adiponectin, and interleukin-10 (IL-10)) correlated positively with body mass index (BMI) [6].

There is a continuous linear relationship between BP and cardiovascular outcome, even before the BP threshold for hypertension diagnosis is reached [7], emphasising the need for accurate and early identification of increased BP. Twenty-four-hour (24 h) ambulatory blood pressure monitoring (ABPM) is regarded as a gold standard measure to identify hypertension [8]. Twenty-four-hour ambulatory BP is also a stronger predictor of cardiovascular events, target end-organ damage [8], and mortality [7] when compared to clinic BP. It is therefore recommended to use 24 h ABPM for accurate out-of-office and early diagnosis of hypertension, and it was additionally suggested to monitor BP in individuals with obesity [9].

To the best of our knowledge, no studies investigated different BP modalities and their relationships with various inflammatory markers in young overweight-to-obese (OW/OB) adults. We firstly aimed to compare BP, inflammatory, and lifestyle measures between normal weight and OW/OB groups. Secondly, to explore the relationships of clinic and 24 h ambulatory BP with leptin, IL-6, IL-8, TNF-α, adiponectin, IL-10, C-reactive protein (CRP), and an integrated inflammatory score in young adults with OW/OB, while considering visceral adiposity index.

## Methods

### Study population

This study used cross-sectional data from the African Prospective study on the Early Detection and Identification of Cardiovascular disease and Hypertension (African-PREDICT) [10]. We used pre-existing data where individuals provided written informed consent before participation. Volunteers resided in or around the Potchefstroom area and were sporadically recruited and tested from 2013 to 2017. Inclusion criteria for the screening phase included normal clinic BP (<140/90 mmHg), normal fasting blood glucose (≤5.6 mmol/L) and/or glycated haemoglobin (HbA1c) (<6.5%), normal tympanic temperature (≤37.5 °C), no self-reported chronic disease or treatment thereof, not infected with the human immunodeficiency virus, pregnant, or breastfeeding. The original African-PREDICT study included *n* = 1202 participants. In this study, we excluded participants with incomplete anthropometric data (*n* = 1) and/or those who used anti-inflammatory medication (*n* = 7). We included *n* = 1194 participants (*n* = 619 women and *n* = 575 men of *n* = 603 Black and *n* = 591 White ethnicity), between 20–30 years of age. This study was approved by the Health Research Ethics Committee of the North-West University (NWU-00247-21-A1) and complies with the Helsinki Declaration for medical research on human participants.

### Anthropometric- and total energy expenditure measurements

A trained anthropometrist used standardised methods of the International Society for Advancement of Kinanthropometry to obtain anthropometric measures [11]. Weight (kg) (SECA 813 Electronic scale, SECA, Hamburg, Germany), and height (cm) (SECA 213 Stadiometer, SECA, Hamburg, Germany) were measured to calculate BMI (weight (kg)/height (m^2^)). Waist circumference (WC) was measured as part of a sex-specific visceral adiposity index which served as an indirect measure of visceral adiposity [12]. Visceral adiposity index for men = (WC / 39.68 + [1.88 × BMI]) × (triglycerides / 1.03) × (1.31 / high-density lipoprotein cholesterol; and visceral adiposity index for women = (WC / 36.58 + [1.89 × BMI]) × (triglycerides/0.81) × (1.52 / high-density lipoprotein cholesterol) [12]. Total energy expenditure (TEE) was measured with a chest-worn Actiheart accelerometer device (CamNtech Ltd., England, UK) over seven consecutive days and was divided by each participant’s weight (kg).

### Cardiovascular measurements

Before the first clinic BP measurement, the participant relaxed for 5 minutes in the seated position. Clinic BP was measured with an appropriately sized GE Critikon latex-free Dura-Cuff in conjunction with a Dinamap^®^ Procare Blood Pressure Monitor (GE Medical Systems, Milwaukee, WI, USA) while the participant was seated with the arm to be measured at heart level. Clinic BP measurements were taken twice on each arm, first on the left arm and then on the right arm, followed by a 5-minute interval, and again on the right and then the left arm. Systolic blood pressure (SBP) and diastolic blood pressure (DBP) were captured during each measurement and the mean value of the four readings was used for subsequent analysis.

A validated 24 h ABPM (Cardio(X)plore® CE120, Meditech, Budapest, Hungary) [13] was fitted on the non-dominant arm of the participant to be measured, by use of an appropriately sized cuff. The ABPM was fitted to each participant at the same time every day, in the late morning hours. The apparatus was programmed to take recordings every 30 minutes throughout the day (06h00 to 22h00) and every hour throughout the night (22h00 to 06h00). Instructions were given to participants on how to ensure successful inflations across the 24 h measurement period. Data were downloaded and CardioVisions (Meditech, Budapest, Hungary) ABPM Software was used to determine day and night, SBP and DBP levels. For 24 h ambulatory BP measurements, only 3.18% of participants had <60% valid 24 h ambulatory BP measurements. The average successful inflation rate of this study was 88%.

### Blood sampling and biochemical analyses

Samples were stored in a biofreezer at −80 °C until analysed. Leptin, adiponectin, IL-6, and TNF-α levels were measured from serum with an enzyme-linked immunosorbent assay (ELISA) kit (R&D systems, Minneapolis, MN, USA), and analysed on a Synergy H4 hybrid microplate reader (BioTek, Winooski, VT, USA). A MILLIPEX Map Human High Sensitivity T Cell Magnetic Bead Panel (EMD Millipore, Merck, Missouri, USA) was used to measure IL-10 and IL-8. This panel was analysed using LuminexMAP technology on the Luminex 200TM analyser which performed immunoassays on the surface of MagPlex-C microspheres, fluorescent-coded magnetic beads.

The integrated inflammatory score [6] was calculated by dividing each of the cytokines and adipokines into tertiles, with the lowest tertile assigned a score of 1, to the highest tertile assigned a score of 3. The integrated inflammatory score was calculated as the sum of the assigned scores from pro-inflammatory cytokines and adipokines (leptin, IL-6, IL-8, and TNF-α), followed by subtraction of the sum of the scores from the anti-inflammatory cytokines and adipokines (adiponectin and IL-10).

Triglycerides (TG), γ-glutamyl transferase (GGT), and CRP were analysed from serum samples (Cobas Integra® 400plus, Roche, Basel, Switzerland). Glycated haemoglobin was analysed in EDTA whole blood (Cobas Integra® 400plus, Roche, Basel, Switzerland) and serum cotinine was analysed on the Immulite (Siemens, Erlangen, Germany) apparatus. Information regarding intra- and inter-assay variability as well as the limit of detection for respective biomarkers used in this study are shown in Supplementary Table 1.

### Statistical analyses

Data were analysed with IBM^®^ SPSS^®^ Statistics Software version 28 (IBM Corporation, Armonk, New York) and graphically presented with GraphPad Prism 5.03 (GraphPad Software, California, USA). The normality of variables was determined through visual inspection of QQ-plots, and the evaluation of skewness and kurtosis coefficients.

Participants were divided into normal weight and OW/OB groups according to BMI with normal weight classified as BMI < 25 kg/m^2^. Comparisons between groups for demographic (age, sex, and ethnicity), inflammatory- (leptin, IL-6, IL-8, TNF-α, adiponectin, IL-10, the integrated inflammatory score, and CRP), BP- (clinic SBP, DBP, and 24 h ambulatory SBP, DBP), and lifestyle measures (cotinine, GGT, and TEE) were evaluated with analysis of covariance, adjusted for age, sex, and ethnicity. Categorical differences were compared by means of a Chi-square test. Partial correlations were performed between BP measures and inflammatory markers with adjustments for age, sex, and ethnicity, in normal weight and OW/OB groups. Backward linear regression analyses were performed between clinic and ambulatory BP and inflammatory markers, in normal weight and OW/OB groups. Analyses were repeated for day-time and night-time ambulatory BP (results not shown). Covariates included age, sex, ethnicity, cotinine, GGT, and TEE, and an additional analysis also included the visceral adiposity index. Sensitivity analyses to test for the effect of nocturnal dipping status, clinic heart rate, and hyperleptinemia, respectively, on the association of 24 h ambulatory BP with inflammatory markers. In the OW/OB group, partial correlations of BMI with inflammatory markers and BP measures were determined with adjustments for age, sex, and ethnicity. The Williams *t*-test was used to compare the strength of correlations between BMI and the different inflammatory markers, as well as BMI and different BP modalities.

## Results

Characteristics of the normal weight and OW/OB groups are presented in Table 1. Of the total group, 43.3% were OW/OB of which 70.0% were hyperleptinemics, and comprised of 45.1% men, 47.2% Black individuals, with a median age of 25.00 ± 3.18 years. Table 1 illustrates the comparison of BP measures, inflammatory markers, and other lifestyle and biochemical markers between the normal weight and OW/OB groups. The OW/OB group had higher clinic SBP, DBP, and 24 h SBP, DBP (all *p* < 0.001), a higher inflammatory score, leptin, IL-6, TNF-α, and CRP with lower adiponectin and IL-8 (all *p* ≤ 0.036). The OW/OB group also showed higher HbA1c, TG, and GGT levels with lower TEE (all *p* < 0.001) compared to the normal weight group.

**Table 1:** Comparison of cardiovascular, inflammatory, and lifestyle measures in normal weight and overweight-to-obese groups

	Body mass index (kg/m^2^)		
	Normal weight	Overweight-to-obese	
	(<25 kg/m^2^)	(≥25 kg/m^2^)	*p*-trend
*n*	677	517	
**Demographic factors**			
Age	24.03 ± 2.97	25.21 ± 3.18	**<0.001**
Sex, men, *n* (%)	342 (50.5)	233 (45.1)	0.062
Ethnicity, Black, *n* (%)	359 (53.0)	244 (47.2)	**0.046**
**Anthropometric measures**			
Body mass index (kg/m^2^)	21.32 ± 2.18	29.91 ± 4.79	**<0.001**
**Inflammation markers**			
Leptin (ng/mL)	7.24 (0.60; 38.58)	23.99 (4.74; 100.60)	**<0.001**
Interleukin-6 (pg/mL)	0.87 (0.33; 2.89)	1.41 (0.54; 4.53)	**<0.001**
Interleukin-8 (pg/mL)	1.91 (0.50; 7.92)	1.72 (0.41; 6.09)	**0.036**
Tumour necrosis factor-α (pg/mL)	1.03 (1.02; 2.30)	1.11 (0.52; 2.69)	**0.017**
Adiponectin (µg/mL)	5.87 (4.75; 12.96)	3.08 (0.68; 10.53)	**<0.001**
Interleukin-10 (pg/mL)	5.08 (1.00; 22.07)	4.69 (1.04; 18.13)	0.16
C-reactive protein (mg/L)	0.52 (0.06; 5.39)	1.75 (0.21; 14.11)	**<0.001**
Inflammatory score	2.98 (1.00; 6.00)	4.67 (2.00; 8.00)	**<0.001**
**Cardiovascular measurements**			
Clinic systolic blood pressure (mmHg)	116 ± 12	122 ± 11	**<0.001**
Clinic diastolic blood pressure (mmHg)	78 ± 8	80 ± 7	**<0.001**
24 h Systolic blood pressure (mmHg)	114 ± 9	121 ± 9	**<0.001**
24 h Diastolic blood pressure (mmHg)	68 ± 6	70 ± 6	**<0.001**
**Lifestyle and biochemical measures**			
Hyperleptinemia, *n* (%)	193 (28.5)	362 (70.0)	**<0.001**
Total energy expenditure (kCal/kg/day)	34.66 ± 10.27	30.23 ± 4.32	**<0.001**
Cotinine (ng/mL)	143.55 (15.70; 500.04)	130.02 (19.75; 425.21)	0.41
*γ*-glutamyl transferase (U/L)	15.85 (5.50; 46.80)	21.83 (7.80; 73.33)	**<0.001**
Glycated haemoglobin (%)	5.30 ± 0.29	5.35 ± 0.34	**<0.001**
Triglycerides (mmol/L)	0.66 (0.29; 1.52)	0.80 (0.34; 2.07)	**<0.001**

Values are expressed as arithmetic means and standard deviation (for normally distributed data) or geometric means with 5th and 95th percentiles (for non-normally distributed data), or proportions (for categorical data). Groups were compared with analyses of covariance, adjusted for age, sex, and ethnicity. Categorical differences were compared by means of chi-square. Bold values denote statistically significant (*p* < 0.05) differences

Supplementary Table 2, Supplementary Figs. 1, and 2 indicates the relationships between BP and inflammatory markers in the normal weight and OW/OB groups, adjusted for age, sex, and ethnicity. In the OW/OB group only, clinic SBP and DBP correlated with leptin (both *r* ≤ 0.10; *p* ≤ 0.024). Twenty-four-hour SBP and DBP correlated with leptin in the OW/OB group (both *r* ≤ 0.22; *p* < 0.001) with a modest correlation in the normal weight group (both *r* ≤ 0.08; *p* ≤ 0.039). In the OW/OB group, 24 h SBP correlated with IL-6 (*r* = 0.12; *p* = 0.006) and the inflammatory score (*r* = 0.12; *p* = 0.005), while 24 h DBP correlated with TNF-α (*r* = 0.14; *p* = 0.001), IL-8 (*r* = -0.09; *p* = 0.033), and the inflammatory score (*r* = 0.16; *p* ≤ 0.001). In both the normal weight and OW/OB groups, 24 h SBP correlated with TNF-α (both *r* ≤ 0.10; *p* ≤ 0.022), while both 24 h SBP and DBP showed a weak correlation with CRP (*r* ≤ 0.13; *p* ≤ 0.037). In the normal weight group only, clinic DBP correlated with IL-10 (*r* = -0.10; *p* = 0.012).

**Fig. 1:**
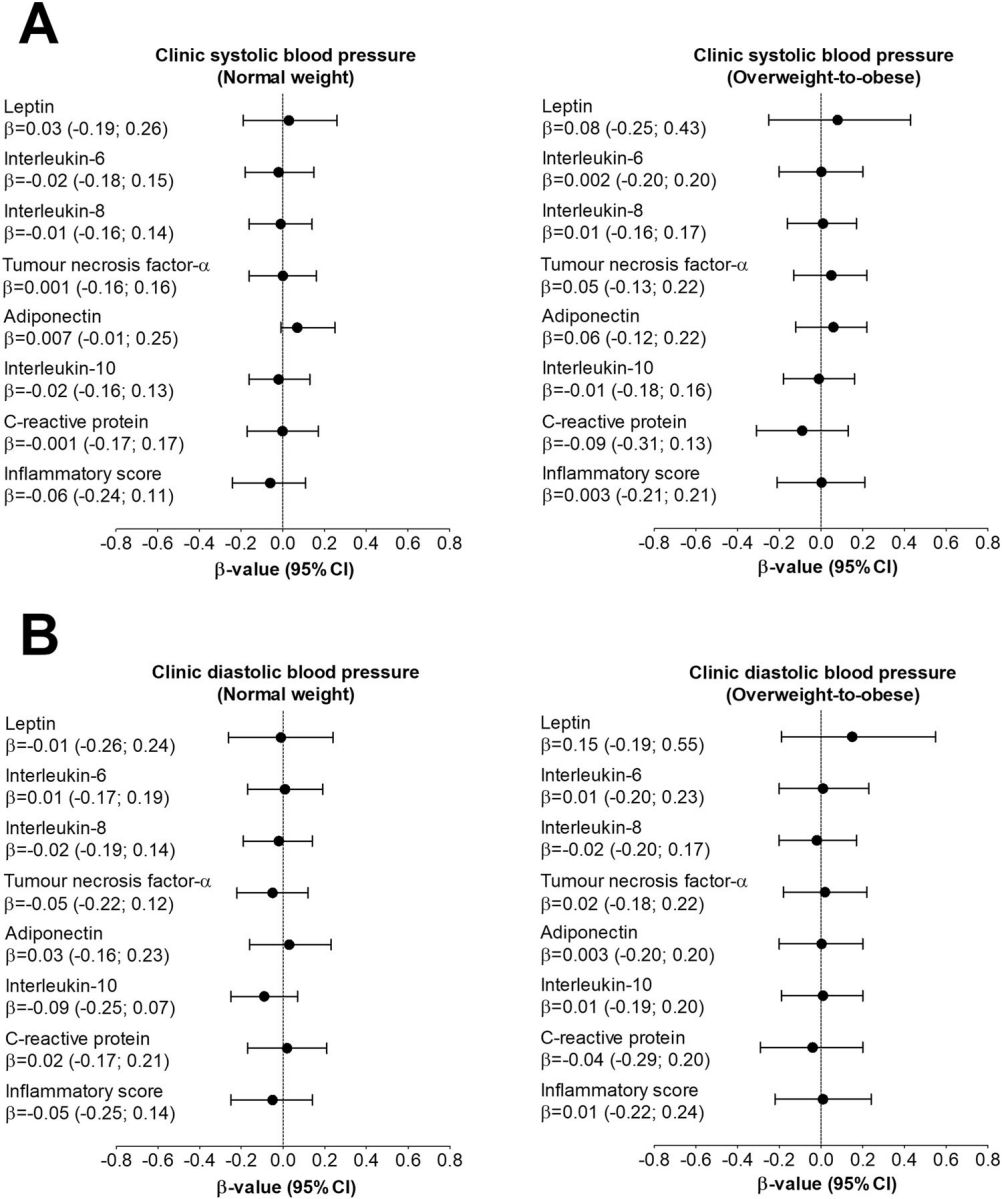
Associations between inflammatory markers and clinic blood pressure in the normal weight and overweight-to-obese groups. Models were adjusted for age, sex, ethnicity, cotinine, γ-glutamyl transferase, total energy expenditure, and visceral adiposity index. *P*-value < 0.05 was regarded as statistically significant

**Fig. 2:**
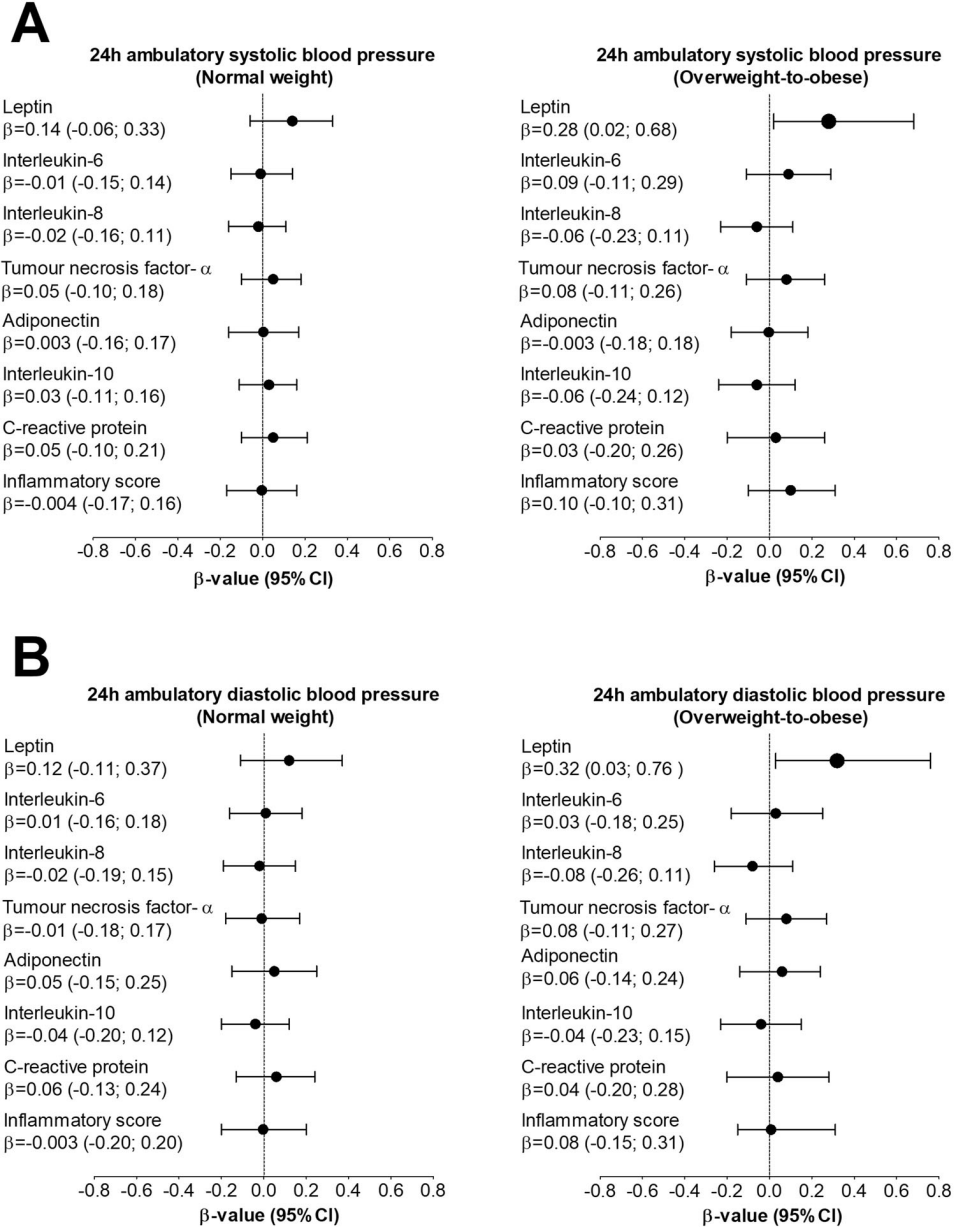
Associations between inflammatory markers and 24 h ambulatory blood pressure in the normal weight and overweight-to-obese groups. Models were adjusted for age, sex, ethnicity, cotinine, γ-glutamyl transferase, total energy expenditure, and visceral adiposity index. *P*-value < 0.05 was regarded as statistically significant

Multivariable adjusted backward linear regression analyses of BP measures with inflammatory measures, independent of age, sex, ethnicity, cotinine, GGT, and TEE are shown in Supplementary Tables 3.1, 3.2. In the OW/OB group only, 24 h SBP (*β* = 0.28; *p* = 0.035) and 24 h DBP (*β* = 0.32; *p* = 0.034) associated with leptin. The same model was repeated with an additional adjustment for visceral adiposity index in Supplementary Table 4, Figs. 1, and 2, and the results remained the same. No associations were evident between clinic BP and any other inflammatory markers. No associations were found between day-time or night-time ambulatory BP and any inflammatory markers (results not shown). Sensitivity analysis showed that nocturnal dipping status and clinic heart rate, respectively, did not change our main results. In a sensitivity analysis, the OW/OB group was additionally divided into normoleptinemic and hyperleptinemic groups which showed a positive association of 24 h SBP (*β* = 0.38; *p* = 0.005) and 24 h DBP (*β* = 0.31; *p* = 0.038) with leptin in the hyperleptinemic participants with OW/OB only.

Supplementary Table 5 shows partial correlations in the OW/OB group after adjustments for age, sex, and ethnicity. Body mass index correlated with leptin (*r* = 0.56; *p* < 0.001), IL-6 (*r* = 0.38; *p* < 0.001), TNF-α (*r* = 0.12; *p* = 0.005), adiponectin (*r* = -0.22; *p* < 0.001), IL-10 (*r* = -0.12; *p* = 0.008), CRP (*r* = 0.38; *p* < 0.001), the inflammatory score (*r* = 0.34; *p* < 0.001), clinic SBP (*r* = 0.13; *p* = 0.003), 24 h SBP (*r* = 0.39; *p* < 0.001), and 24 h DBP (*r* = 0.21; *p* < 0.001). The Williams *t*-test revealed that BMI correlated stronger with leptin compared to other inflammatory markers and BMI correlated stronger with 24 h SBP and DBP compared to clinic SBP and DBP, respectively (all *p* < 0.001).

## Discussion

We compared BP measures, inflammatory markers, and lifestyle measures between normal weight and OW/OB groups, and investigated the relationships of both clinic and 24 h ambulatory BP with various inflammatory markers in OW/OB young adults. After adjustment for age, sex and ethnicity, the OW/OB group had higher clinic and 24 h ambulatory BP as well as an overall worse inflammatory profile compared to the normal weight group. In fully adjusted regression models, 24 h SBP and DBP, but not clinic BP associated positively with leptin in the OW/OB group only, and the results remained the same with additional adjustments for visceral adiposity index. Body mass index showed a stronger relationship with leptin when compared to the other inflammatory markers, and BMI showed a stronger relationship with 24 h ambulatory BP when compared to clinic BP.

The OW/OB group of this study had higher clinic- and 24 h ambulatory BP, HbA1c, and TG when compared to the normal weight group. The same group also had higher levels of inflammatory markers, and it is known that a pro-inflammatory state is a driving factor in the onset of hypertension [14]. Only the OW/OB group showed positive associations of 24 h ambulatory SBP and DBP with leptin, independent of age, sex, ethnicity, cotinine, GGT, and TEE. A possible explanation for the positive relationship of leptin with 24 h SBP and DBP, but not clinic BP, may be that 24 h ABPM is regarded as a superior measure to identify hypertension when compared to clinic BP [8]. The superiority of 24 h ABPM compared to clinic BP can be attributed to its ability to differentiate between hypertension phenotypes, the consideration of BP variability over the course of the day, and individual responses to allostatic load [7]. In the participants with OW/OB of this study, additional analysis showed that BMI had a stronger relationship with 24 h SBP and DBP when compared to clinic SBP and DBP, respectively. A study of 63 adolescents with obesity, of which 49.2% were females, with a mean age of 14.0 ± 1.7 years, found that four patients were hypertensive according to clinic BP, whereas 13 patients with normal clinic BP were hypertensive according to 24 h ambulatory BP [9]. The authors stated that reliance on clinic BP alone may result in a third of hypertensive adolescents with obesity being undiagnosed [9], but also mentioned that the small sample size is a limitation of their study [9].

Only leptin, and no other inflammatory markers, showed a positive association with 24 h SBP and DBP in the OW/OB group of this study. It was suggested that excess leptin plays a major role in increased BP among individuals with obesity [15]. We also showed a stronger relationship between BMI and leptin when compared to the other inflammatory markers in our OW/OB group. Another study performed in normoglycemic participants with a high-normal BMI (24.8 ± 2.6) found that 24 h ABPM associated positively with free leptin in both sustained and masked hypertensive individuals [16]. Albeit a small study sample with only *n* = 166 hypertensives and *n* = 66 masked hypertensive participants above 30 years of age, their findings suggest that leptin-related vascular impairment is similar to sustained and masked hypertension. Various mechanisms play a role in the leptin-induced adiposity-related pathogenesis of increased BP. High levels of leptin may induce the synthesis of endothelin-1 which leads to endothelial dysfunction [17]. Chronically elevated leptin will also result in a loss of nitric oxide- and endothelium-derived hyperpolarising factor-mediated vasodilation [18]. Leptin additionally binds to neurons involved in sympathetic nervous system activation [15], contributing to increased peripheral resistance [18]. Decreased blood flow to the kidney then activates the renin-angiotensin-aldosterone system which results in sodium and water reabsorption, and ultimately increased BP [15].

Mutations of the leptin gene resulting in leptin deficiency are uncommon in humans; however, such individuals develop severe obesity with low-to-normal BP, inferring that leptin is essential for the progression of adiposity-related increased BP [19]. An opposing argument is that adiposity-related hypertension is not dependent on leptin as BP does not increase with leptin administration in individuals with lipodystrophy (the absence of depot-specific or generalised subcutaneous adipose tissue) or congenital leptin deficiency [20]. It should be noted that low, but sufficient levels of leptin are beneficial as it functions to induce satiety and vasodilation [18]. The patients mentioned in the study by Brown et al., had increased satiety and weight loss with leptin administration [20], suggesting that they possibly did not chronically receive excess amounts of leptin and were therefore not leptin resistant. It is expected that optimal levels of leptin will rather have a beneficial role in which patients respond with normal- or even lower BP [18]. In an obesogenic state, hyperleptinemia (>15 ng/mL) [21] results in selective leptin resistance where individuals lose the beneficial effects of leptin, but the detrimental effects become amplified [22]. The detrimental effects of leptin on BP will only be observed in a setting of hyperleptinemia and/or leptin resistance [22]. In a sensitivity analysis of our study, the OW/OB group was additionally divided into normoleptinemic and hyperleptinemic groups, and 24 h SBP and 24 h DBP positively associated with leptin in the hyperleptinemic individuals with OW/OB only. In a setting of excess adiposity and hyperleptinemia, the detrimental link between 24 h BP and leptin is robust; however, leptin may still have beneficial or compensatory functions which normalise BP in individuals with excess adiposity, but still normal levels of leptin.

It was also found that despite the lack of subcutaneous adipose tissue, and subsequently a lack of leptin, in individuals with lipodystrophy [20], congenital leptin deficiency, or biallelic leptin receptor gene variants [23] some of those individuals still have hypertension. The authors concluded that leptin is not essential for adiposity-related hypertension to occur and that other mechanisms may be involved [23]. Leptin is primarily secreted by subcutaneous adipose tissue, whereas other inflammatory markers, such as IL-6 are 2-to-3 times higher in visceral adipose tissue when compared to subcutaneous adipose tissue [24] and also contribute to hypertension development [25]. Controversial outcomes among different studies on leptin and BP may therefore partly be a result of differences in adipose tissue distribution with different underlying inflammatory mechanisms among various body types.

Against this backdrop, we additionally adjusted for visceral adiposity index in multivariate adjusted associations of 24 h SBP and DBP with leptin in the OW/OB group, and the results remained unchanged. It can be reasoned that this young population has not yet transitioned from predominantly subcutaneous adiposity to visceral adiposity as often observed with ageing [26]. Considering that leptin is primarily secreted by subcutaneous adipose tissue [24], excess subcutaneous adipose tissue explains the relationships of 24 h SBP and DBP with leptin in our OW/OB group. It also explains the lack of relationships after multivariate adjustments between BP and markers from which the secretion may primarily depend on visceral adiposity, such as IL-6 [24] and possibly the inflammatory score. The literature often emphasises the detrimental effects of visceral adipose tissue on inflammation and its relationship with BP and cardiovascular disease [25, 27]. Our results indicate that the detrimental effects of excess subcutaneous adipose tissue on BP should not be undermined, although further research is needed in this regard with the use of advanced measures to accurately discriminate between subcutaneous- and visceral adipose tissue. This study’s findings may help clarify the controversy as to whether adiposity-related increased BP is dependent on leptin. A large number of studies on leptin and BP were in mice [20], but our findings contribute to human-specific studies, especially at ages younger than 30 years.

The results of this study should be viewed in the context of its strengths and limitations. Under controlled conditions, data were collected by trained staff in a well-equipped and professional research setting. This relatively large sample comprised both women and men, of Black and White ethnicity. Our unique young adult population provides the opportunity to identify early signs of cardiovascular disease development, but we could not determine causality as cross-sectional data was used. We also included both clinic and 24 h ambulatory BP monitoring in a young population to identify associations with an array of inflammatory markers early in the cardiovascular disease continuum. Body mass index cannot discriminate between different types of adipose tissue distribution. However, the relationships of adiposity with inflammatory markers and cardiovascular measures were similar for BMI, WC, and waist-to-height ratio in high adiposity groups of a previous study on the same population [28]. We made use of data from objective lifestyle measures to correct for possible human error in self reported lifestyle measures.

## Conclusion

Twenty-four-hour ambulatory, but not clinic BP, already associates with leptin in young adults with OW/OB, but without overt cardiovascular disease. Hyperleptinemia may indicate early cardiovascular risk linked with elevated BP in young adults with OW/OB from predominantly excess subcutaneous adipose tissue.

### Supplementary information

Supplementary material

Supplementary Figure Legend

Supplementary Figure 1

Supplementary Figure 2
