# Supplementary material for: Twenty-four-hour ambulatory, but not clinic blood pressure associates with leptin in young adults with overweight or obesity: The African-PREDICT study
Source: Hypertens Res. 2023 Oct 23;47(2):478–86. doi: 10.1038/s41440-023-01477-7 (PMC10838765; doi:10.1038/s41440-023-01477-7)
Supplement: Supplementary file 1 — Supplementary material [file 41440_2023_1477_MOESM1_ESM.docx]

**Supplementary material**

**Supplementary Table 1.** Intra- and inter-assay variabilities as well as the limit of detection for respective biomarkers.

| Biomarker | Sample matrix | Intra-assay variability  (CV) | Inter-assay variability  (CV) | Limit  of  detection | Apparatus |
| --- | --- | --- | --- | --- | --- |
| Cotinine | Serum | 10.7 | 14.67 | 2 ng/mL | Immulite 1000 (Siemens, Erlangen, Germany) |
| γ-glutamyltransferase | Serum | 1.8 | 1.8 | 3 U/L | Cobas Integra® 400plus (Roche, Basel, Switzerland) |
| C-reactive protein | Serum | 1.3 | 3.5 | 0.1 mg/L |  |
| Triglycerides | Serum | 1.6 | 1.9 | 0.1 mmol/L |  |
| Glycated haemoglobin | EDTA whole blood | 1.3 | 8.0 | 0.18 mmol/L |  |
| Leptin | Serum | 3.1 | 2.87 | 7.8 pg/mL | ELISA kit (R&D systems, Minneapolis, MN, USA) & Synergy H4 hybrid microplate reader (BioTek, Winooski, VT, USA)  Catalogue nr: Leptin: DLP00; Adiponectin: DRP300; Interleukin-6: HS600B; TNF-α: HSTA00E |
| Adiponectin | Serum | 3.92 | 8.6 | 0.891 ng/mL |  |
| Interleukin-6 | Serum | 7.8 | 9.6 | 0.039 pg/mL |  |
| TNF-α | Serum | 8.7 | 10.4 | 0.022 pg/mL |  |
| Interleukin-8 | Serum | <5% | <15% | 0.13 pg/mL | MILLIPEX Map Human High Sensitivity T Cell Magnetic Bead Panel (EMD Millipore, Merck, Missouri, USA) Catalogue nr: HSTCMAG-28SK |
| Interleukin-10 | Serum | <5% | <20% | 0.56 pg/mL |  |

*Abbreviations: CV, coefficient of variance; LDL-c, low-density lipoprotein cholesterol; HDL-c, high-density lipoprotein cholesterol; and TNF-α, tumour necrosis factor-α.*

**Supplementary Table 2.** Partial correlations of clinic blood pressure and twenty-four-hour ambulatory blood pressure with inflammatory markers in normal weight and overweight-to-obese groups.

|  | Normal weight  (BMI: <25kg/m^2^) | | Overweight-to-obese  (BMI: ≥25kg/m^2^) | |
| --- | --- | --- | --- | --- |
|  | *n*=619 | | *n*=575 | |
|  | *r* | *p* | *r* | *p* |
| ***Clinic systolic blood pressure (mmHg)*** | | | | |
| Leptin (ng/mL) | 0.04 | 0.37 | **0.10** | **0.024** |
| Interleukin-6 (pg/mL) | -0.02 | 0.62 | 0.03 | 0.56 |
| Interleukin-8 (pg/mL) | -0.02 | 0.54 | 0.002 | 0.97 |
| Tumour necrosis factor-α (pg/mL) | 0.03 | 0.43 | 0.08 | 0.09 |
| Adiponectin (µg/mL) | 0.05 | 0.24 | 0.02 | 0.69 |
| Interleukin-10 (pg/mL) | -0.02 | 0.55 | -0.01 | 0.77 |
| C-reactive protein (mg/L) | 0.03 | 0.48 | -0.02 | 0.69 |
| Inflammatory score | -0.05 | 0.20 | 0.04 | 0.35 |
| ***Clinic diastolic blood pressure (mmHg)*** | | | | |
| Leptin (ng/mL) | 0.002 | 0.95 | **0.15** | **<0.001** |
| Interleukin-6 (pg/mL) | <0.001 | 1.00 | 0.03 | 0.53 |
| Interleukin-8 (pg/mL) | -0.03 | 0.42 | -0.02 | 0.61 |
| Tumour necrosis factor-α (pg/mL) | -0.03 | 0.43 | 0.07 | 0.14 |
| Adiponectin (µg/mL) | 0.02 | 0.53 | -0.03 | 0.45 |
| Interleukin-10 (pg/mL) | -**0.10** | **0.012** | 0.001 | 0.99 |
| C-reactive protein (mg/L) | 0.04 | 0.32 | 0.02 | 0.67 |
| Inflammatory score | -0.03 | 0.37 | 0.07 | 0.11 |
| ***Twenty-four-hour systolic blood pressure (mmHg)*** | | | | |
| Leptin (ng/mL) | **0.11** | **0.004** | **0.22** | **<0.001** |
| Interleukin-6 (pg/mL) | -0.01 | 0.76 | **0.12** | **0.006** |
| Interleukin-8 (pg/mL) | -0.04 | 0.35 | -0.08 | 0.080 |
| Tumour necrosis factor-α (pg/mL) | **0.09** | **0.022** | **0.10** | **0.021** |
| Adiponectin (µg/mL) | -0.04 | 0.32 | -0.05 | 0.27 |
| Interleukin-10 (pg/mL) | 0.02 | 0.55 | -0.07 | 0.14 |
| C-reactive protein (mg/L) | **0.10** | **0.012** | **0.10** | **0.018** |
| Inflammatory score | 0.04 | 0.28 | **0.12** | **0.005** |
| ***Twenty-four-hour diastolic blood pressure (mmHg)*** | | | | |
| Leptin (ng/mL) | **0.08** | **0.039** | **0.28** | **<0.001** |
| Interleukin-6 (pg/mL) | 0.01 | 0.90 | 0.07 | 0.11 |
| Interleukin-8 (pg/mL) | -0.03 | 0.50 | -**0.09** | **0.033** |
| Tumour necrosis factor-α (pg/mL) | 0.03 | 0.46 | **0.14** | **0.001** |
| Adiponectin (µg/mL) | 0.02 | 0.61 | -0.02 | 0.63 |
| Interleukin-10 (pg/mL) | -0.04 | 0.33 | -0.05 | 0.25 |
| C-reactive protein (mg/L) | **0.08** | **0.037** | **0.13** | **0.003** |
| Inflammatory score | 0.03 | 0.51 | **0.16** | **<0.001** |

Adjusted for age, sex, and ethnicity. Bold values denote statistically significant (p<0.05) correlations. *Abbreviation: BMI, body mass index.*

**Supplementary Table 3.1** Association between inflammatory markers and clinic blood pressure in normal weight and overweight-to-obese groups.

|  | Normal weight  (BMI: <25kg/m^2^) | | |  | Overweight-to-obese  (BMI: ≥25kg/m^2^) | | |
| --- | --- | --- | --- | --- | --- | --- | --- |
|  | Adj. R^2^ | β (95%CI) | *p*-value |  | Adj. R^2^ | β (95%CI) | *p*-value |
| ***Clinic systolic blood pressure (mmHg)*** | | | | | | | |
| Leptin  (ng/mL) | 0.30 | 0.04  (-0.18; 0.26) | 0.72 |  | 0.21 | 0.09  (-0.22; 0.44) | 0.51 |
| Interleukin-6  (pg/mL) | 0.29 | -0.01  (-0.17; 0.15) | 0.93 |  | 0.19 | 0.01  (-0.18; 0.21) | 0.89 |
| Interleukin-8  (pg/mL) | 0.29 | -0.01  (-0.17; 0.14) | 0.84 |  | 0.18 | 0.004  (-0.16; 0.17) | 0.97 |
| Tumour necrosis factor-α (pg/mL) | 0.29 | 0.01  (-0.15; 0.16) | 0.95 |  | 0.21 | 0.06  (-0.11; 0.23) | 0.50 |
| Adiponectin  (µg/mL) | 0.31 | 0.05  (-0.11; 0.23) | 0.48 |  | 0.20 | 0.03  (-0.14; 0.19) | 0.77 |
| Interleukin-10  (pg/mL) | 0.29 | -0.02  (-0.17; 0.13) | 0.81 |  | 0.19 | -0.01  (-0.18; 0.16) | 0.90 |
| C-reactive protein  (mg/L) | 0.29 | 0.01  (-0.16; 0.18) | 0.92 |  | 0.21 | -0.07  (-0.28; 0.14) | 0.52 |
| Inflammatory score | 0.31 | -0.06  (-0.24; 0.11) | 0.45 |  | 0.20 | 0.02  (-0.18; 0.22) | 0.82 |
| ***Clinic diastolic blood pressure (mmHg)*** | | | | | | | |
| Leptin  (ng/mL) | 0.13 | -0.004  (-0.25; 0.24) | 0.97 |  | 0.06 | 0.15  (-0.19; 0.55) | 0.33 |
| Interleukin-6  (pg/mL) | 0.13 | 0.01  (-0.17; 0.19) | 0.91 |  | 0.03 | 0.02  (-0.20; 0.23) | 0.89 |
| Interleukin-8  (pg/mL) | 0.13 | -0.02  (-0.19; 0.14) | 0.77 |  | 0.04 | -0.02  (-0.20; 0.17) | 0.86 |
| Tumour necrosis factor-α (pg/mL) | 0.13 | -0.05  (-0.22; 0.12) | 0.56 |  | 0.04 | 0.02  (-0.17; 0.21) | 0.82 |
| Adiponectin  (µg/mL) | 0.13 | 0.03  (-0.16; 0.23) | 0.69 |  | 0.03 | -0.01  (-0.19; 0.18) | 0.94 |
| Interleukin-10  (pg/mL) | 0.14 | -0.09  (-0.25; 0.07) | 0.26 |  | 0.03 | 0.01  (-0.19; 0.20) | 0.94 |
| C-reactive protein  (mg/L) | 0.13 | 0.02  (-0.16; 0.21) | 0.79 |  | 0.04 | -0.03  (-0.28; 0.20) | 0.77 |
| Inflammatory score | 0.13 | -0.05  (-0.25; 0.14) | 0.58 |  | 0.03 | 0.02  (-0.21; 0.24) | 0.87 |

Backward multiple regression analyses were adjusted for age, sex, ethnicity, cotinine, *γ*-glutamyl transferase, and total energy expenditure. Bold values denote statistically significant (p<0.05) associations. *Abbreviations: Adj. R^2^, adjusted R-square; BMI, body mass index, and CI, confidence interval.*

**Supplementary Table 3.2** Association between inflammatory markers and twenty-four-hour ambulatory blood pressure in normal weight and overweight-to-obese groups.

|  | Normal weight  (BMI: <25kg/m^2^) | | |  | Overweight-to-obese  (BMI: ≥25kg/m^2^) | | |
| --- | --- | --- | --- | --- | --- | --- | --- |
|  | Adj. R^2^ | β (95%CI) | *p*-value |  | Adj. R^2^ | β (95%CI) | *p*-value |
| ***Twenty-four-hour systolic blood pressure (mmHg)*** | | | | | | | |
| Leptin  (ng/mL) | 0.33 | 0.14  (-0.06; 0.33) | 0.17 |  | **0.25** | **0.28**  **(0.02; 0.68)** | **0.035** |
| Interleukin-6  (pg/mL) | 0.31 | 0.01  (-0.14; 0.15) | 0.95 |  | 0.23 | 0.10  (-0.09; 0.30) | 0.30 |
| Interleukin-8  (pg/mL) | 0.32 | -0.02  (-0.16; 0.11) | 0.74 |  | 0.23 | -0.07  (-0.23; 0.11) | 0.47 |
| Tumour necrosis factor-α (pg/mL) | 0.32 | 0.05  (-0.09; 0.19) | 0.49 |  | 0.23 | 0.09  (-0.09; 0.27) | 0.33 |
| Adiponectin  (µg/mL) | 0.32 | -0.02  (-0.17; 0.14) | 0.83 |  | 0.22 | -0.03  (-0.20; 0.15) | 0.76 |
| Interleukin-10  (pg/mL) | 0.32 | 0.02  (-0.11; 0.15) | 0.76 |  | 0.22 | -0.06  (-0.24; 0.12) | 0.51 |
| C-reactive protein  (mg/L) | 0.32 | 0.06  (-0.09; 0.21) | 0.45 |  | 0.22 | 0.05  (-0.17; 0.27) | 0.66 |
| Inflammatory score | 0.32 | 0.02  (-0.14; 0.17) | 0.83 |  | 0.24 | 0.13  (-0.06; 0.33) | 0.17 |
| ***Twenty-four-hour diastolic blood pressure (mmHg)*** | | | | | | | |
| Leptin  (ng/mL) | 0.08 | 0.12  (-0.11; 0.37) | 0.30 |  | **0.10** | **0.32**  **(0.03; 0.76)** | **0.034** |
| Interleukin-6  (pg/mL) | 0.07 | 0.01  (-0.16; 0.19) | 0.88 |  | 0.07 | 0.06  (-0.16; 0.27) | 0.61 |
| Interleukin-8  (pg/mL) | 0.05 | -0.02  (-0.19; 0.15) | 0.83 |  | 0.08 | -0.08  (-0.26; 0.11) | 0.41 |
| Tumour necrosis factor-α (pg/mL) | 0.06 | -0.002  (-0.18; 0.17) | 0.98 |  | 0.08 | 0.12  (-0.07; 0.30) | 0.22 |
| Adiponectin  (µg/mL) | 0.07 | 0.03  (-0.16; 0.23) | 0.71 |  | 0.04 | 0.01  (-0.18; 0.20) | 0.90 |
| Interleukin-10  (pg/mL) | 0.08 | -0.04  (-0.20; 0.12) | 0.63 |  | 0.06 | -0.04  (-0.24; 0.15) | 0.66 |
| C-reactive protein  (mg/L) | 0.08 | 0.06  (-0.13; 0.24) | 0.53 |  | 0.07 | 0.07  (-0.16; 0.31) | 0.54 |
| Inflammatory score | 0.06 | 0.01  (-0.19; 0.20) | 0.94 |  | 0.08 | 0.11  (-0.11; 0.34) | 0.30 |

Backward multiple regression analyses were adjusted for age, sex, ethnicity, cotinine, *γ*-glutamyl transferase, and total energy expenditure. Bold values denote statistically significant (p<0.05) associations. *Abbreviations: Adj. R^2^, adjusted R-square; BMI, body mass index, and CI, confidence interval.*

**Supplementary Table 4.** Association between inflammatory markers and twenty-four-hour ambulatory blood pressure in normal weight and overweight-to-obese groups according to body mass index after additional adjustments for visceral adiposity index.

|  | Normal weight  (BMI: <25kg/m^2^) | | |  | Overweight-to-obese  (BMI: ≥25kg/m^2^) | | |
| --- | --- | --- | --- | --- | --- | --- | --- |
|  | Adj. R^2^ | β (95%CI) | *p*-value |  | Adj. R^2^ | β (95%CI) | *p*-value |
| ***Twenty-four-hour systolic blood pressure (mmHg)*** | | | | | | | |
| Leptin  (ng/mL) | 0.33 | 0.14  (-0.06; 0.33) | 0.17 |  | **0.25** | **0.28**  **(0.02; 0.68)** | **0.035** |
| ***Twenty-four-hour diastolic blood pressure (mmHg)*** | | | | | | | |
| Leptin  (ng/mL) | 0.08 | 0.12  (-0.14; 0.37) | 0.30 |  | **0.10** | **0.32**  **(0.03; 0.76)** | **0.034** |

Backward multiple regression analyses were adjusted for age, sex, ethnicity, cotinine, *γ*-glutamyl transferase, total energy expenditure, and visceral adiposity index. Bold values denote statistically significant (p<0.05) associations. *Abbreviations: Adj. R^2^, adjusted R-square; BMI, body mass index, and CI, confidence interval.*

**Supplementary Table 5.** Partial correlation of body mass index with inflammatory markers and blood pressure measures in the overweight-to-obese group.

|  | Overweight-to-obese group  (BMI: ≥25kg/m^2^) | | |
| --- | --- | --- | --- |
|  | Body mass index | | |
|  | Partial correlations | | Williams t-test |
|  | *r* | *p* | *p-*value |
| ***Inflammatory markers*** | | | |
| Leptin (ng/mL) | 0.56 | **<0.001** | **<0.001** |
| Interleukin-6 (pg/mL) | 0.38 | **<0.001** |  |
| Leptin (ng/mL) | 0.56 | **<0.001** | **<0.001** |
| Interleukin-8 (pg/mL) | -0.07 | 0.12 |  |
| Leptin (ng/mL) | 0.56 | **<0.001** | **<0.001** |
| Tumour necrosis factor-α (pg/mL) | 0.12 | **0.005** |  |
| Leptin (ng/mL) | 0.56 | **<0.001** | **<0.001** |
| Adiponectin (µg/mL) | -0.22 | **<0.001** |  |
| Leptin (ng/mL) | 0.56 | **<0.001** | **<0.001** |
| Interleukin-10 (pg/mL) | -0.12 | **0.008** |  |
| Leptin (ng/mL) | 0.56 | **<0.001** | **<0.001** |
| C-reactive protein (mg/L) | 0.38 | **<0.001** |  |
| Leptin (ng/mL) | 0.56 | **<0.001** | **<0.001** |
| Inflammatory score | 0.34 | **<0.001** |  |
| ***Blood pressure measures*** | | | |
| Clinic systolic blood pressure (mmHg) | 0.13 | **0.003** | **<0.001** |
| 24h Systolic blood pressure (mmHg) | 0.39 | **<0.001** |  |
| Clinic diastolic blood pressure (mmHg) | 0.07 | 0.12 | **<0.001** |
| 24h Diastolic blood pressure (mmHg) | 0.21 | **<0.001** |  |

Adjusted for adjusted for age, sex, and ethnicity. Bold values denote statistically significant (p<0.05) correlations. *Abbreviation: BMI, body mass index.*
