# Supplementary material for: Twenty-four-hour ambulatory, but not clinic blood pressure associates with leptin in young adults with overweight or obesity: The African-PREDICT study
Source: Hypertens Res. 2023 Oct 23;47(2):478–86. doi: 10.1038/s41440-023-01477-7 (PMC10838765; doi:10.1038/s41440-023-01477-7)
Supplement: Supplementary file 2 — Supplementary Figure Legend [file 41440_2023_1477_MOESM2_ESM.docx]

**Supplementary Figure 1**: Associations between inflammatory markers and clinic blood pressure in the normal weight and overweight-to-obese groups. Models were adjusted for age, sex, and ethnicity. *P*-value <0.05 was regarded as statistically significant.

**Supplementary Figure 2**: Associations between inflammatory markers and 24h ambulatory blood pressure in the normal weight and overweight-to-obese groups. Models were adjusted for age, sex, and ethnicity. *P*-value <0.05 was regarded as statistically significant.
