# Supplementary figures and images for: Twenty-four-hour ambulatory, but not clinic blood pressure associates with leptin in young adults with overweight or obesity: The African-PREDICT study
Source: Hypertens Res. 2023 Oct 23;47(2):478–86. doi: 10.1038/s41440-023-01477-7 (PMC10838765; doi:10.1038/s41440-023-01477-7)

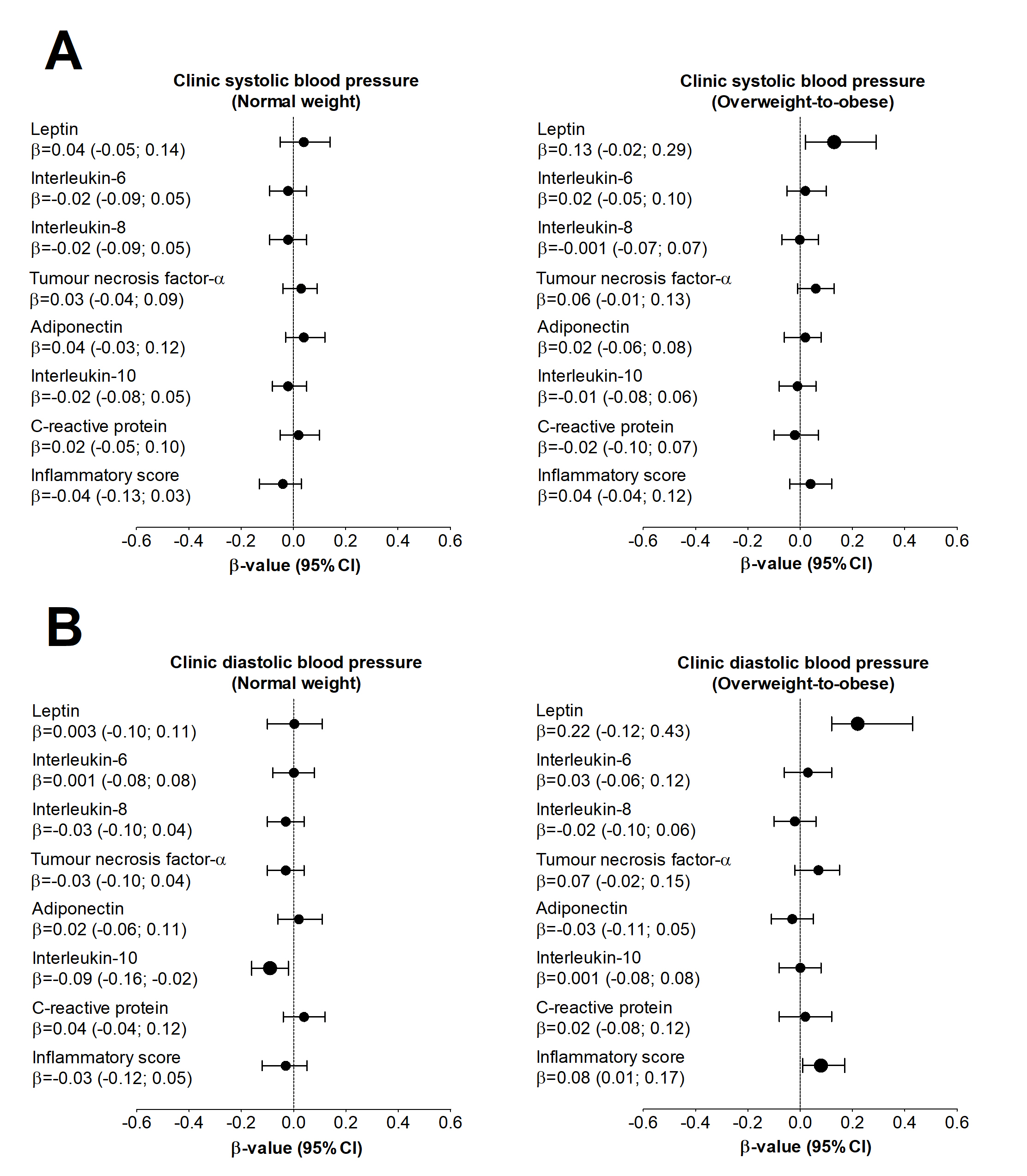

Supplement: Supplementary file 3 — Supplementary Figure 1 [file 41440_2023_1477_MOESM3_ESM.jpg]

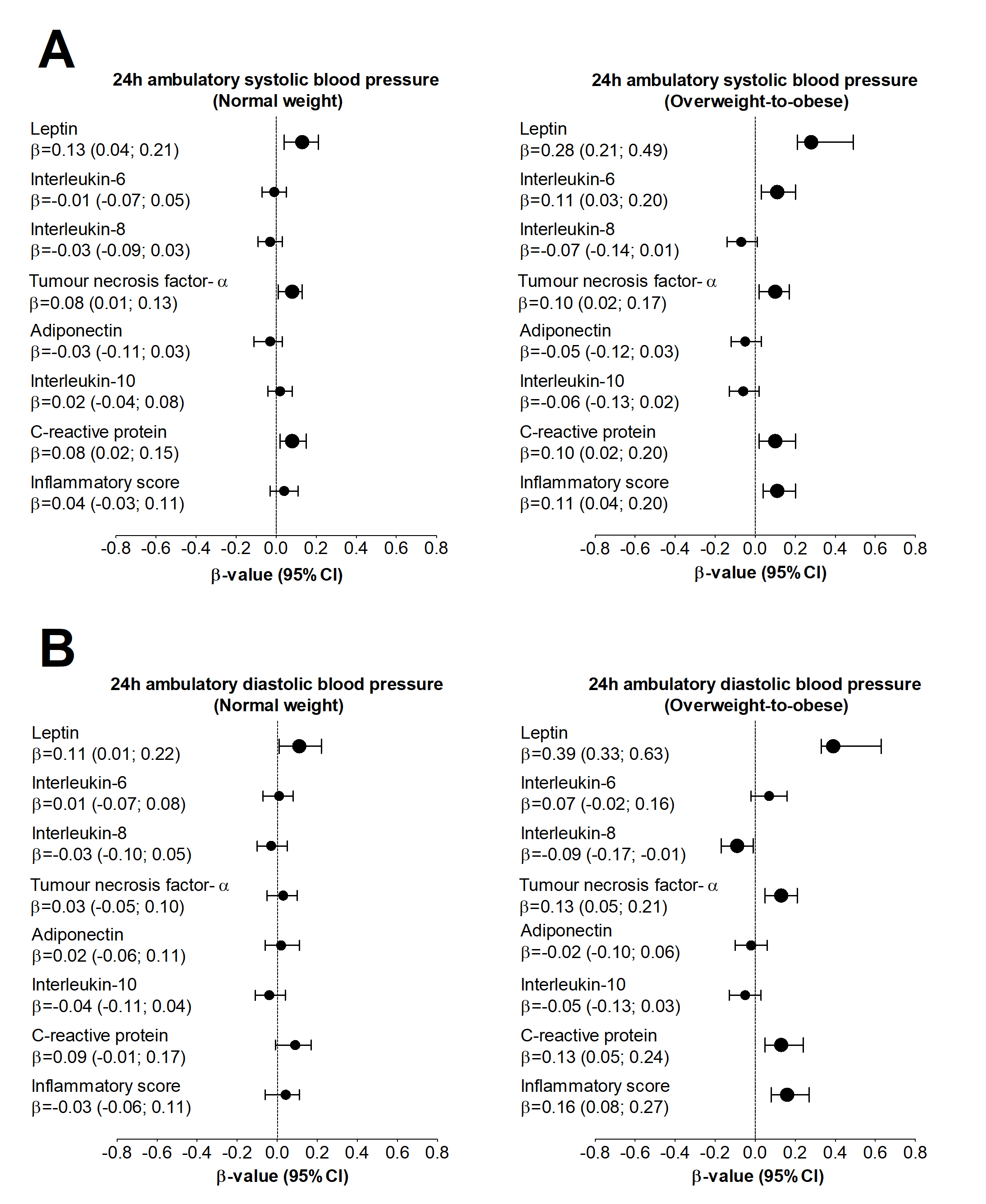

Supplement: Supplementary file 4 — Supplementary Figure 2 [file 41440_2023_1477_MOESM4_ESM.jpg]
